# Parliament and the Profession

**Published:** 1917-03-24

**Authors:** 


### PARLIAMENT AND THE PROFESSION.

### Central Medical Benefit Fund.

Sir Edwin Cornwall informed Mr. Chancellor that the total sum paid into the pool representing the Central Medical Benefit Fund is arrived at according to the contribution cards surrendered by approved societies, and not by computing the actual number of stamps sold.

### Cerebral Meningitis at Reading.

Mr. Macpherson stated that inquiries are being made, and the facts will be communicated to the House of Commons, in respect of an outbreak of cerebral meningitis, referred to in a question by Captain Douglas Hall, in a building at Coley, near Reading, at present occupied by the School of Technical Training, Royal Flying Corps.

### Medical Students and Military Service.

Mr. Hancock asked the Under-Secretary of State for War, in view of the probable shortage of fully-qualified physicians and surgeons after the war, for information as to the position of medical students on attaining military age. He was informed by Mr. Macpherson that medical students are not called up if they are within twenty-four months of their medical degree or licence, provided they are enrolled in an O.T.C.; if they are not within this period they are not called to the Colours if, being under thirty-one, they are classified lower than B 1.

### State Registration of Nurses.

Mr. Hayes Fisher, replying for the Prime Minister, informed Major Chapple that a number of Nursing Associations mentioned by him have expressed opinions in favour of the State registration of nurses, but that the information before him did not show that they are in agreement as to the character of the scheme. Legislation on the subject could not at the present time be promised.

### Local Authorities and Venereal Diseases.

Mr. Hayes Fisher informed Mr. Rowntree that twenty local authorities in England have already provided facilities for the free treatment of these diseases in twenty-eight hospitals. The Local Government Board have approved the schemes of twenty-four other authorities, and these arrangements will be working shortly. In addition, the Board have under consideration further schemes submitted by forty-four local authorities.

